# Dietary sodium and potassium intake in people with diabetes: are guidelines being met?

**DOI:** 10.1038/s41387-020-0126-5

**Published:** 2020-06-17

**Authors:** Sara Baqar, Adrian Michalopoulos, George Jerums, Elif I. Ekinci

**Affiliations:** 1 Department of Endocrinology, Austin Health, Heidelberg, VIC Australia; 2 Department of Medicine, The University of Melbourne, Parkville, VIC Australia; 3 Department of Medicine, Austin Health, Heidelberg, VIC Australia

**Keywords:** Diabetes, Nutrition

## Abstract

**Objective:**

Despite public health bodies advocating for lowering dietary sodium and increasing potassium intake to improve cardiovascular outcomes, people with diabetes are not meeting these targets. We hypothesize that (i) both at an individual level and within the cohort, there will be a low adherence to the guidelines and (ii) sodium and potassium intake will remain stable over time.

**Methods:**

We conducted this prospective study in a cohort of 904 participants with diabetes who provided 24-h urine collections from 2009 to 2015. Dietary sodium and potassium intake were estimated from 24-h urinary sodium (uNa) and potassium (uK) measurements. Additional data were collected for: 24-h urinary volume (uVol), creatinine (uCr),; serum creatinine, urea, estimated glomerular filtration rate (eGFR), glycated haemoglobin (HbA1c), fasting glucose, lipids); clinical characteristics (age, blood pressure (BP), body mass index (BMI) and duration of diabetes). Adherence to recommended dietary sodium (uNa < 2300 mg/24 h (100mmol/24 h)) and potassium (uK > 4680 mg/24 h(120 mmol/24)) intake were the main outcome measures.

**Results:**

Participants (*n* = 904) completed 3689 urine collections (average four collections/participant). The mean ± SD (mmol/24 h) for uNa was 181 ± 73 and uK was 76 ± 25. After correcting uNa for uCr, 7% and 5% of participants met dietary sodium and potassium guidelines respectively. Males were less likely to meet sodium guidelines (OR 0.40, *p* < 0.001) but were more likely to meet potassium guidelines (OR 6.13, *p* < 0.001). Longer duration of diabetes was associated with higher adherence to sodium and potassium guidelines (OR 1.04, *p* < 0.001 and OR 0.96, *p* = 0.006 respectively). Increasing age was significantly associated with adherence to potassium guidelines (OR 0.97, *p* = 0.007).

**Conclusions:**

People with diabetes do not follow current dietary sodium and potassium guidelines and are less likely to change their dietary intake of sodium and potassium over time.

## Introduction

Cardiovascular related diseases are the leading cause of morbidity and mortality^1^, especially in those with diabetes^2^. Blood pressure is a modifiable cardiovascular risk factor^1^. Dietary sodium and potassium intake play a pivotal role in blood pressure regulation^1,3^. High dietary salt intake can raise blood pressure^3,4^ whereas a diet low in sodium and high in potassium is associated with lower blood pressure^5,6^. Not surprisingly, public health bodies such as the American Diabetes Association^7^ and Institute of Medicine^8^, recommend an upper limit of sodium intake at 2300 mg per day (100 mmol/24 h)^7,8^ and daily potassium intake of 4680 mg per day (120 mmol/24)^8^.

However, worldwide mean sodium intake is almost double the recommended level of intake (3950 mg/24 h (172 mmol/24 h))^9^. A recent meta-analysis of the Australian population found that the mean sodium intake in people with type 1 or type 2 diabetes was 9.66 g/24 h (420 mmol/24 h)^10^. Therefore, almost all of the population will have to implement major dietary changes in order to achieve the recommended targets^11^. Whether adhering to these guidelines are necessary and achievable^12^ for people with diabetes has been called into question. Despite its blood pressure lowering effects, lower sodium intake has been paradoxically associated with higher cardiovascular and all-cause mortality in people with diabetes^13,14^. Moreover, in a community-dwelling population, inverse associations have been demonstrated between low sodium intake and myocardial infarction and mortality^15^.

The association between low dietary sodium intake and total and cardiovascular mortality has been inconsistent. Study findings have suggested lower sodium intake is associated with either a higher risk of death^13–15^, a lower risk of death^16–18^, U-/J-shaped relationship^19,20^, an uncertain association^21,22^ or no association^23^ between sodium intake and cardiovascular health outcomes. Methodological differences may account for these discrepancies. In earlier studies, sodium intake was estimated by dietary recall which underestimates intake by 50%^24^. Measurement of 24-h urinary sodium excretion (uNa)^25^ provides a more accurate estimation of dietary sodium intake.

Whilst four studies in Australia have utilized 24 h urine collections to assess adherence to dietary sodium and potassium intake in people with diabetes^12,26–28^, only one study has assessed dietary sodium and potassium consumption in people with both type 1 and type 2 diabetes^26^. We, therefore, aimed to provide an updated estimate of whether people with diabetes of any type are adhering to current dietary sodium and potassium guidelines. We assessed our study cohort’s sodium and potassium dietary intake over the study period (cohort level analysis). Additionally, we estimated the percentage of adherence to these dietary guidelines over the study period (individual-level analysis). We hypothesize that (i) there will be an overall low adherence to the guidelines and (ii) sodium and potassium intake will not change over time.

## Research design and methods

### Study design and recruitment of participants

In this prospective study, we recruited 904 participants with a diagnosis of diabetes from our university teaching hospital diabetes outpatient clinics. We assessed adherence to dietary sodium and potassium guidelines using 24-h urinary excretion values, as the best estimate for dietary intake, from 2009 to 2015 inclusive. The dietary guidelines used to define sodium and potassium intake for this study were based on the American Diabetes Association recommendations^7^ of sodium intake <2300 mg per day (100 mmol/24 h)^7,8^ and from the Institute of Medicine potassium intake recommendations at >4680 mg per day (120 mmol/24)^8^ as they are specifically intended for high-risk subgroups, such as those with diabetes. This study was approved by the Austin Health Human Research Ethics Committee. Written consent was obtained from all participants.

### Endpoints

The primary endpoints were to (i) provide an estimate of an individuals’ sodium intake over the seven-year period and to (ii) assess the adherence to the dietary sodium intake guidelines at a cohort level. Our secondary endpoints were to estimate the potassium intake, as above, at both the individual and cohort level.

### Biochemical urinary and serum analyses

Twenty-four-hour urinary sodium (uNa), urinary potassium (uK), urinary volume (uVol), urinary creatinine (uCr), urinary urea (uUrea), and urinary glucose (uGlu) were recorded for each urine sample collected by the participants over the seven-year period. Biochemical parameters of serum creatinine, estimated glomerular filtration rate (eGFR) as calculated by Chronic Kidney Disease Epidemiology Collaboration (CKD-EPI) formula, serum urea, uric acid, glycated haemoglobin (HbA1c), fasting glucose, total cholesterol, high-density lipoprotein (HDL), low-density lipoprotein (LDL), triglycerides and c-peptide were collected.

uVol was recorded by weighing the specimen. No preservative was used for the 24 h urine collection. From January 2009 to January 2012, uNa, uK and uGlu, uCr, fasting glucose, serum urea, total cholesterol, serum HDL and serum triglycerides were analyzed by Beckman Coulter UniCel DXC800⁄ 600 System(s) Analyzer. From January 2012 until December 2015 our institution’s pathology department protocol was changed and the same parameters were analyzed on Roche Cobas 8000. The HbA1c was measured by immunoassay on Roche Integra and LDL was calculated using the Friedewald equation^29^.

Given ~90% of ingested sodium and 80% of potassium is excreted in the urine^25^, we used 24-h urinary excretion to most accurately estimate dietary sodium and potassium intake over other methods such as dietary recall^24^. We have previously demonstrated that a single measurement of 24-h UNa can predict habitual dietary sodium intake in people with type 2 diabetes, with an intra-individual coefficient of variation 21 ± 1% suggesting day-to-day variation of sodium intake is approximately 20%^12^. To improve accuracy, we analyzed multiple urine collections for each participant.

### Ensuring the accuracy of urine collections

Patients attending the diabetes clinics routinely perform 24-h urine collections. Written and verbal instructions are provided by clinicians to ensure the accuracy of urine collections. For each participant’s uNa collection, we corrected for any changes in renal function over time and for the possibility of incomplete urine collections by calculating the mean 24 hUCr measurements and then utilizing the following formula:

$${\it{\mathrm{Corrected}}}\;{\it{\mathrm{uNa} = }}\frac{{{\it{\mathrm{uNa}}}\;{\it{x}}\;{\it{\mathrm{overall}}}\;{\it{\mathrm{mean}}}\;{\it{\mathrm{uCr}}}\;{\it{\mathrm{for}}}\;{\it{\mathrm{that}}}\;{\it{\mathrm{individual}}}}}{{{\it{\mathrm{uCr}}}\;{\it{in}}\;{\it{\mathrm{that}}}\;{\it{\mathrm{sample}}}}}$$

To remain comparable with previous regional surveys and blood pressure trials^13,14,19,20^, whereby uNa levels were measured, we did not adjust dietary sodium and potassium intake excretion to compensate for insensible losses. The uNa and uK values were then used to determine the adherence to the dietary guidelines at both an individual and a population level and to determine the intra-individual variability of sodium and potassium intake over time.

### Anthropometry and clinical characteristics

Where available, additional characteristics such as blood pressure, body mass index (BMI) and duration of diabetes were recorded.

### Statistics and data analysis

A single investigator collected all the data. A 10% random validity check was done to assess for accuracy. Baseline characteristics are reported as mean ± standard deviation. Yearly averages of both 24 h uNa and uK excretion were calculated and graphed accordingly to assess the cohort level adherence. Individual-level adherence was assessed by estimating the percentage a participant would adhere to the dietary guidelines over time. The intra-individual change over time was defined as an average of the percentage that each participant met the guidelines. For example, if the participant had ten 24 h uNa readings and met the guidelines once, their percentage would be 10%. We then calculated the mean of these percentages for the cohort and represented the data as median (interquartile range). The data were represented as a box-plot. This was deemed the most effective method of calculating the intra-individual change over the study period time. Univariate logistic regression analysis was used to describe the univariate relationships between explanatory variables such as age, sex, duration of diabetes, HbA1c, fasting glucose, eGFR, serum urea, and lipid profile and a patient’s ability to meet the dietary sodium and potassium guidelines. The effect of these explanatory variables on an individual’s ability to meet the guidelines was reported with an odds ratio and a 95% confidence interval. Multivariate logistic regression analysis was performed determining the effect of age, sex, duration of diabetes and HbA1c on the ability to meet the guidelines. Multiple regression results allow us to account for potential redundancy in the explanatory variables. Co-linearity tests were performed to account for any substantial correlation between any of the variables included in the multivariate analysis that may have affected the results. A *p*-value of <0.05 was considered statistically significant. Statistical analysis was performed with STATA version 14.1 software (StataCorp. 2015. *Stata Statistical Software: Release 14*. College Station, TX: StataCorp LP).

## Results

### Baseline cohort characteristics

This study included 904 participants of whom 732 (81%) had type 2 diabetes, 144 (15.9%) had type 1 diabetes and 10 (1.1%) had latent autoimmune diabetes in adults. Secondary causes such as corticosteroid-induced diabetes, new-onset diabetes after transplant, hemochromatosis, and pancreatitis induced pancreatic insufficiency were seen in 12 (1.3%) participants. Six participants had no documentation of their type of diabetes.

Participants completed a total of 3689 24 h urine collections, ranging from one to sixteen collections per participant with an average of four 24 h urine collections. Baseline characteristics are summarized in Table 1. Once adjusted for uCr excretion the corrected mean ± SD sodium excretion (mmol/24 h) was 181 ± 73 and the mean ± SD potassium excretion (mmol/24h) was 76 ± 25 (Table 1). After this correction, 63 of the 904 (7%) participants were adherent to the dietary sodium guidelines and 42 of the 904 participants (5%) participants were adherent to the dietary potassium guidelines. The mean ± SD sodium to potassium ratio was calculated separately and in our cohort was 2.5 ± 0.9.

**Table 1:** Baseline characteristics of study participants.

Variable	Mean ± SD	95% Confidence interval
Total number of participants	904	
Male sex	551	
Age (years)	60 ± 13	
Duration of diabetes (years)	13 ± 9	−5–32
Body Mass Index (kg/m^2^)	31.4 ± 6.7	18.0–44.7
Systolic blood pressure (mmHg)	134 ± 16	103–165
Diastolic blood pressure (mmHg)	73 ± 10	54–93
24 h uNa (mmol/24 h)	181.2 ± 72.7	35.9–326.6
24 h uK (mmol/24 h)	76.1 ± 25.4	25.4–126.8
24 h uCr (mmol/24 h)	12.8 ± 4.4	4.0–21.5
24 h uGlu (mmol/24 h)	52.0 ± 104.6	−157.3–261.2
24 h uUrea (mmol/24 h)	441.2 ± 156.8	128–755
HbA1c (%), [mmol/mol]	7.7 ± 1.2, [61]	5.3–10.0
Fasting Glucose (mmol/l)	8.5 ± 2.6	3.4–13.7
CKD-EPI eGFR (ml/min/1.73m^2^)	77.9 ± 23.9	30.0–125.6
Cholesterol (mmol/l)	4.1 ± 0.9	2.3–5.9
HDL (mmol/l)	1.2 ± 0.7	−0.1–2.6
LDL (mmol/l)	2.2 ± 0.8	0.7–3.7
Triglycerides (mmol/l)	1.6 ± 1.1	−0.5–3.8

Data are expressed as mean ± standard deviation with 95% confidence interval.

*IQR* interquartile range, *24* *h uNa* 24-h urinary sodium excretion, *24* *h uK* 24-h urinary potassium excretion, *24* *h uCr* 24-h urinary creatinine excretion, *24* *h uGlu* 24-h urinary glucose excretion, *24* *h uUrea* 24-h urinary urea excretion, *CKD-EPI* Chronic Kidney Disease Epidemiology Collaboration, *eGFR* estimated glomerular filtration rate estimated using the CKD-EPI equation.

The following classes of medications, which are known to affect sodium and or potassium excretion, were taken at the time of the urine collections (*n* = 3689) as follows: Sodium-glucose co-transporter-2 inhibitors in 13 (0.4%) collections; Potassium Sparing Diuretics in 80 (2.2%) collections; Loop diuretics in 273 (2.2%) collections and Thiazide diuretics in 663 (18%) collections.

### Cohort level analysis

**Fig. 1:**
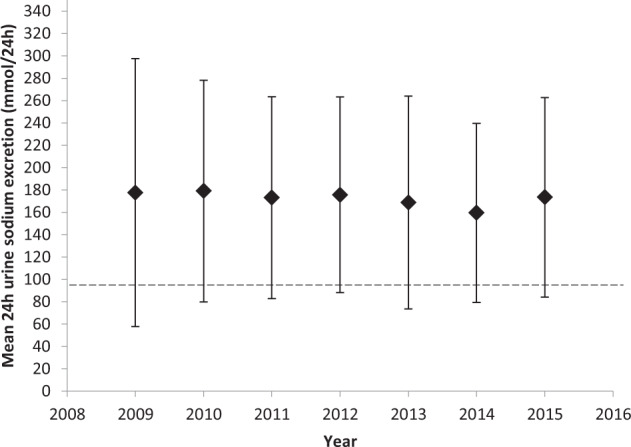
Mean yearly urinary sodium excretion of total cohort from 2009–2015. Results are presented as mean +/− standard deviation. The horizontal dashed line represents the dietary sodium intake target as per the American Diabetes Association guidelines < 100 mmol/24 h^7^.

**Fig. 2:**
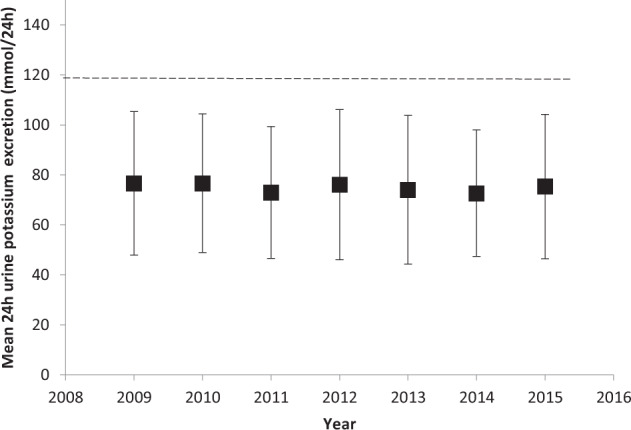
Mean yearly urinary potassium excretion of total cohort from 2009–2015. Results are presented as mean +/− standard deviation. The horizontal dashed line represents the dietary potassium intake target as per the Institute of Medicine guidelines > 120 mmol/24 h^8^.

The mean yearly uNa from 2009–2015 remained above the recommended sodium intake target of <100 mmol/24 h (Fig. 1)^30^. Similarly, the mean yearly uK remained well below the potassium guideline recommendations of >120 mmol/24 h (Fig. 2)^31^. Over time, at a population level, sodium and potassium intake did not change (Figs. 1 and 2).

### Individual-level analysis

The intra-individual variability and the percentage of participants adhering to the guidelines over time remained low. The percentage of adherence to the guidelines over time was (median (interquartile range) (number of times guidelines met / number of 24hUNa samples)) 0% (25%) for sodium guidelines and 0% (0%) for potassium guidelines.

**Fig. 3:**
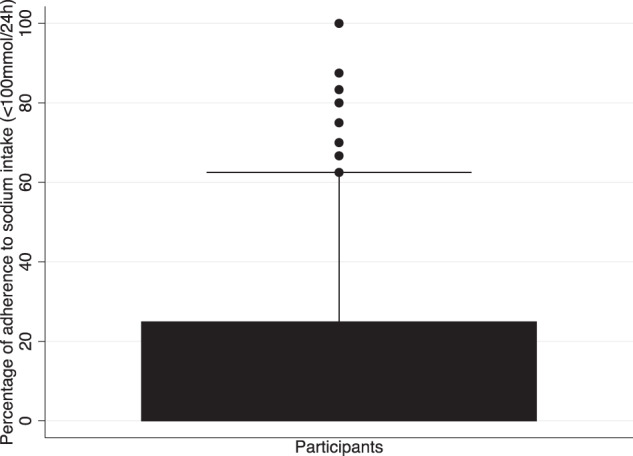
Adherence to the dietary sodium intake guidelines at an individual level. Box plot representing a participant’s likelihood to adhere to the dietary sodium guidelines over the seven-year period. The box-plot suggests that 50% of patients are not likely to adhere to the sodium intake guidelines and of the remaining 50%, 50% are only likely to adhere to the guidelines ~20% of the time and the other 50% adhere to the guidelines ~60% of the time.

**Fig. 4:**
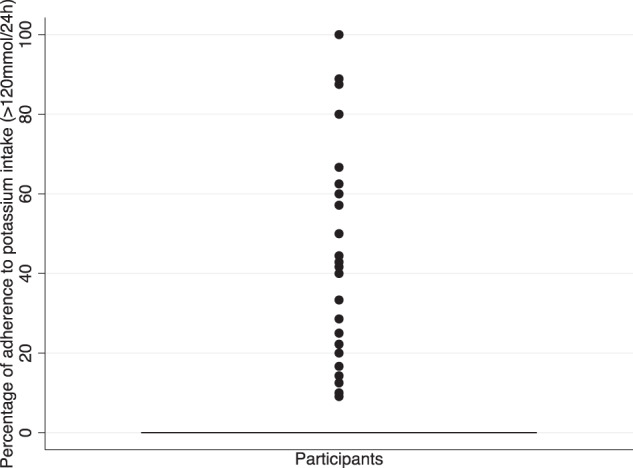
Adherence to the dietary potassium intake guidelines at an individual level. Box plot representing a participant’s likelihood to adhere to the potassium intake guidelines over the seven-year period. The box-plot suggests that 100% of participants are unlikely to adhere to the potassium intake guidelines.

Figure 3^32^ represents the participants’ percentage of adhering to the dietary sodium guidelines. The box-plot suggests that 50% of participants are non-adherent. Of the remaining 50%, 50% adhere to the guidelines ~20% of the time and 50% ~60% of the times. Figure 4^33^ represents the participants’ likelihood of adhering to dietary potassium guidelines. The box-plot suggests that 100% of participants are unlikely to adhere to the potassium intake guidelines.

### Univariate analysis

We examined the likelihood of participants meeting the guidelines in relation to eleven explanatory variables (age, sex, duration of diabetes, HbA1c, fasting glucose, eGFR, serum urea and lipid profile (cholesterol, HDL, LDL and triglycerides)) (Table 2).

**Table 2:** Results of univariate and multivariate analysis.

Explanatory variable	Urinary sodium guidelines						Urinary potassium guidelines					
	Univariate			Multivariate			Univariate			Multivariate		
	OR	95% CI	*p*-value	OR	95% CI	*p*-value	OR	95% CI	*p*-value	OR	95% CI	*p*-value
Age (years)	1.01	(1.00–1.02)	0.06	1.00	(0.98–1.01)	0.54	0.97	(0.96–1.00)	**0.007**	0.99	(0.96–1.00)	0.21
Sex (males)	**0.40**	**(0.31–0.52)**	**<0.001**	**0.38**	**(0.28–0.53)**	**<0.001**	**6.13**	**(3.39–11.1)**	**<0.001**	**5.73**	**(2.91–11.3)**	**<0.001**
Duration of diabetes (years)	**1.04**	**(1.02–1.05)**	**<0.001**	**1.04**	**(1.02–1.05)**	**<0.001**	0.96	(0.93–0.99)	**0.006**	0.98	(0.95–1.01)	0.12
HbA1c (%)	0.93	(0.84–1.04)	0.19	**0.86**	**(0.76–0.97)**	**0.01**	0.77	(0.63–0.94)	0.009	**0.77**	**(0.62–0.96)**	**0.02**
Fasting glucose (mmol/l)	0.97	(0.94–1.01)	0.15				0.95	(0.89–1.01)	0.13			
CKD_EPI eGFR (ml/min/1.73 m^2^)	**0.99**	**(0.99–1.00)**	**0.003**				1.01	(1.00–1.02)	0.008			
Serum urea (mmol/l)	1.01	(0.98–1.05)	0.48				0.96	(0.88–1.04)	0.33			
Cholesterol (mmol/l)	0.90	(0.79–1.03)	0.13				1.08	(0.86–1.35)	0.53			
HDL (mmol/l)	1.00	(0.95–1.06)	0.9				0.88	(0.51–1.53)	0.65			
LDL (mmol/l)	0.86	(0.73–1.00)	0.05				1.03	(0.78–1.35)	0.85			
Triglycerides (mmol/l)	**0.81**	**(0.71–0.92)**	**0.002**				1.06	(0.90–1.25)	0.51			

Data are expressed as odds ratio, 95% confidence interval and *p*-value. The univariate logistic regression analyses was performed on 11 variables as potential predictors of adhering to dietary guidelines. The multivariate analysis was performed on four variables. Bold values indicate statistical significance.

*OR* odds ratio, *CKD-EPI* Chronic Kidney Disease Epidemiology Collaboration, *eGFR* estimated glomerular filtration rate estimated using the CKD-EPI equation.

Regarding sodium intake, males were less likely to meet the guidelines (OR 0.40, *p* < 0.001). Participants were more likely to meet guidelines as duration of diabetes increased (OR 1.04, *p* < 0.001). Age had no significant effect (OR 1.01, *p* = 0.06). A higher eGFR and triglyceride level was associated with participants being less likely to meet guidelines (OR 0.99, *p* = 0.003, and OR 0.86, *p* = 0.04 respectively). Higher LDL levels were associated with a trend towards a lower likelihood of participants meeting guidelines (OR 0.86, *p* = 0.05). No other parameters had any significant effect (Table 2).

For potassium intake, male participants were more likely to meet the guidelines (OR 6.13, *p* < 0.001). Increasing duration of diabetes (OR 0.96, *p* = 0.006) and age had a higher likelihood of adhering to the guidelines (OR 0.97, *p* = 0.007). No other parameters had any significant effect (Table 2).

### Multivariate analysis

The following variables: age, sex, duration of diabetes and HbA1c, were entered into a multivariate regression analysis as these showed the strongest association with a participant’s ability to adhere to the dietary sodium and potassium guidelines from the univariate analysis. For dietary sodium, male sex (OR 0.38, *p* < 0.001), duration of diabetes (OR 1.04, *p* < 0.001) and HbA1c (OR 0.86, *p* = 0.01) were all significantly associated with a participant’s ability to meet the sodium guidelines when controlled for each other (Table 2). For the dietary potassium guidelines, male sex (OR 5.73, *p* < 0.001) and HbA1c (OR 0.77, *p* = 0.02) showed a significant association when controlled for the other explanatory variables (Table 2). The potential for collinearity was accounted for when these four parameters were placed in the multiple regression analysis and no major correlations were found.

## Discussion

### Key findings

Public health bodies advocate for high sodium and low potassium intake to improve cardiovascular health outcomes. We demonstrated that people with diabetes are having difficulties adhering to these recommendations with 93% and 95% of participants not meeting sodium and potassium guidelines respectively. The likelihood of changing dietary intake over time to adhere to the guidelines is low.

### People with diabetes are not meeting recommended dietary sodium and potassium guidelines and this is unlikely to change over time

The World Health Organisation aims to reduce the mean population sodium intake by 30% by the year 2025^34^. Many countries, including Australia, have signed up to these global targets. Despite strategies being implemented to reduce dietary salt intake over many years^35^, progress has been slow^36,37^. In the present study, we demonstrated that the yearly mean sodium and potassium intake in people with diabetes in Australia, who attended a university teaching hospital, exceeds recommendations made by public health bodies. Only 7% of participants achieved dietary sodium targets (<100 mmol/24 h) and 5% of participants achieved dietary potassium targets (>120 mmol/24 h). These results are consistent with previous studies in people with diabetes^12^ and comparable to other significant past^38,39^ and recent^40^ large-scale population studies whereby the majority of people do not adhere to guidelines.

Additionally, the likelihood of each participant meeting the dietary guidelines over time was low. Our results are in keeping with a recent meta-analysis that demonstrated there had been no change in dietary sodium consumption in the Australian population since 1989^10^. A limitation of that study^10^, however, was that the analysis of change over time was not based on repeat representative samples of the population. An advantage of the current study is having the average of four urine samples per participant. This provides a more accurate measure of temporal changes in dietary intake over time at an individual level.

In the present study, participants were more likely to adhere to the dietary sodium guidelines as the duration of diabetes increased. Previous studies have shown that people with poorer health and comorbidities have reduced appetites and consume less dietary sodium^41^. Interestingly, in our study, as age increased, there was no significant association in the ability to adhere to sodium guidelines. Similarly, in a meta-analysis, there was no relationship between age and sodium intake^10^.

The HbA1c, as a single explanatory variable, had no association with the ability to adhere to sodium intake guidelines. However, in the multivariate analysis, after adjusting for age, sex, and duration of diabetes, lower HbA1c was significantly associated with the ability to meet guidelines. Given the observational nature of the study it is not possible to infer the nature of this relationship.

Males were less likely to adhere to sodium intake guidelines but were more likely to adhere to potassium intake guidelines. Males consume more food than women and as a result have higher sodium as well as higher potassium intakes overall. Our findings are consistent with the recent meta-analysis of Australian sodium consumption demonstrating a sex difference in sodium intake^10^.

### Salt appetite, salt taste perception and sodium content in foods may explain variations in sodium intake

Overall, people with diabetes may have increased salt appetite and reduced salt taste perception to account for the inability to adhere to low sodium intake. It is not known how salt appetite is derived, however, evidence suggests that genetics^42^, environment^43^ and pre-existing disease states^41^ may play a role. A gene polymorphism (C825T) has been associated with low sodium intake in people with type 2 diabetes^42^. People with type 2 diabetes with low sodium intake were found to have the genetic polymorphism, whereas those with high sodium intake did not^42^. Furthermore, people with diabetes and hypertension have reduced salt taste perception and are unlikely to recognize the amount of sodium consumed^44^. No previous study has examined salt appetite and salt taste perception in conjunction with 24 h uNa in people with diabetes. This presents an opportunity for future studies to investigate this concept further.

Sources of dietary sodium are also important to consider. The Japanese diet, for example, despite its high sodium intake, has been associated with lower risk of cardiovascular disease^45^ as dietary sodium comes from soybean products, fish, seaweed and green tea which may have unique properties to benefit the cardiovascular system^45^. In Western countries, such as the United Kingdom, United States^43^ and Australia^46^, ~80% of sodium^46^ is from processed foods. Strategies in Western countries aimed at modifying consumer behaviour, such as advising people not to add salt to their foods, may not reduce dietary sodium intake, as added salt is only a small proportion of their daily sodium intake^47^. Education on sodium content in food may have more bearing^36^ on health outcomes.

However, sodium content in foods is poorly understood by people with type 2 diabetes^28,48^. In a randomized controlled trial, a single session of food label education to help choose low sodium products (<120 mg/100 g) in people with type 2 diabetes did not reduce mean 24 h uNa excretion as compared with placebo, despite high levels of self-reported adherence^28^. Furthermore, in a separate study, despite 80% of people reporting reading food labels, dietary sodium intake, as estimated from 24 h uNa, remained high at 3887 +/− 736 mg/24 h (169 +/− 32 mmol/24 h) in males and 2645 +/− 621 mg/24 h (115 +/− 27 mmol/24 h) in females^48^. The major contributors to dietary sodium intake in people with type 2 diabetes^28^ come from staple foods, such as bread and cereals. Most people are unaware these foods contain large amounts of sodium^48^. Therefore, whilst we did not formally assess the sources of dietary sodium in our study, the aforementioned studies^28,48^ provide insight into the dietary habits of people with diabetes in Australia and may, in part, explain why our cohort did not meet dietary sodium recommendations.

### Potassium intake and the role of the sodium to potassium ratio

Only 5% of participants met dietary potassium intake guidelines. People with diabetes consume processed foods with high sodium contents^46^ which are most likely depleted of potassium^41^. Males were more likely to meet guidelines consistent with previous studies demonstrating that whilst potassium intake is low globally^6,41^, a sex difference for potassium exists^41^. Potassium is considered to play a crucial role in cardiovascular health outcomes and deserves similar attention to sodium intake. Potassium intake has been shown to have an inverse relationship with blood pressure^6^, with those in the highest range of potassium intake having the lowest blood pressure^49^.

The ratio of sodium to potassium intake is considered more important on cardiovascular health outcomes than each factor alone^41,50^. Lower sodium to potassium ratio is associated with lower blood pressure^41^. Higher sodium to potassium ratio^50^, consistent with high sodium intake and low potassium intake, as was seen in our study, is associated with adverse cardiovascular health outcomes^50^.

### Is there a range for optimal sodium intake?

Optimal dietary sodium targets are a matter of ongoing debate. Observational studies demonstrate that sodium intakes below 2645 mg (115 mmol/24 h) and above 4945 mg (215 mmol/24 h) are associated with higher mortality^49,51^. The results of a meta-analysis supports these findings by comparing three sodium groups; high (>5000 mg/24 h or 217 mmol/24 h), moderate (2700–5000 mg/24 h or 117–217 mmol/24 h) and low (<2700 mg/24 h or 117 mmol/24 h), with the risk of a cardiovascular event^20^. Whilst the high sodium group had the highest risk of an event and all-cause mortality, the moderate sodium group was found to be at lower risk of an event and all-cause mortality as compared with the low sodium group^20^. Multiple observational studies report sodium intake between 115 mmol/24 h and 215 mmol/24 h may provide the best cardiovascular outcomes^52^. It is reassuring that the majority of participants in the present study were consuming sodium within this range. Although the Institute of Medicine^8^ and the American Diabetes Association^7^ no longer recommend very low sodium intakes of <70 mmol/24 h in people with diabetes and recommend sodium intake of <100 mmol/24 h consistent with the general population, in light of the aforementioned evidence and the demonstration in our study that people with diabetes are simply unable to meet these targets, these recommendations^7,8^ may need to be further revised. Furthermore, randomized controlled trials assessing sodium intake and hard outcomes such as cardiovascular events and mortality are greatly needed^8^.

### Strengths and limitations

The relatively large sample size of 3689 urine collections is considered a major strength. Furthermore, utilizing 24 h uNa and uK excretion to estimate dietary intake is considered more accurate^25^ over other methods such as dietary recall^24^. The study design being prospective and longitudinal enables the detection of changes in dietary sodium and potassium intake over time and helps in determining the intra-individual variability and the likelihood of change by participants over time. We acknowledge that medications known to affect sodium and or potassium excretion were not excluded. Excluding participants based on the use of these medications, would have significantly reduced the number of participants whose data is available to analyze given these medications are routinely prescribed to people with diabetes. Furthermore we have previously shown that treatment with thiazide diuretics for >4 weeks does not affect uNa excretion^53^. Additionally, the number of urine collections and the time interval between each collection were not consistent between participants. However, a major strength is having an average of four urine collections per participant which more accurately reflects habitual dietary patterns.

Our findings demonstrate that people with diabetes are not adhering to dietary sodium and potassium recommendations at an individual or at a population level. There is a low likelihood of adhering to such guidelines over time as dietary intake of sodium and potassium remains stable. Our findings, coupled with the previous demonstration in observational studies of an association between low dietary sodium intake and adverse cardiovascular health outcomes, questions the necessity for stringent sodium lowering. Long-term interventional studies are greatly needed to determine the ideal yet feasible dietary sodium intake targets in people with diabetes.
